# Supplementary material for: Extracellular Vesicles as Novel Diagnostic and Therapeutic Agents for Non-Melanoma Skin Cancer: A Systematic Review
Source: Int J Mol Sci. 2024 Feb 23;25(5):2617. doi: 10.3390/ijms25052617 (PMC10931606; doi:10.3390/ijms25052617)
Supplement: Supplementary file 1 [file ijms-25-02617-s001.zip › ijms-2831185-supplementary.pdf]

Supplementary Methods

Modified RoB-2 tool. The RoB-2 tool, designed for assessing bias in randomized clinical trials with human subjects, required modifications for application in in vitro research. Adjustments aligned with cell study criteria from a University of Bristol methodology study by Lewis et al.[1]. In the in vitro context, "Randomization" is less directly applicable, as bias often stems from cell line selection and culture conditions. Selection bias arises from systematic differences in chosen cell lines impacting study outcomes. Performance bias was reframed to emphasize intervention variability, crucial for consistent implementation in cell-based research. This shift acknowledges challenges in maintaining standardized interventions in in vitro studies. For detection bias, especially in cell culture studies, "blinding" may be less relevant, so our focus was on outcome evaluation transparency and objectivity. Lastly, assessing other biases involved scrutiny of cell culture techniques, experimental setup, suitable statistical methods, transparency in conflict-of-interest disclosures and funding sources, and data availability for independent verification. The finalised checklist is provided in Table S3.

Quality assessment of the included studies. Each study underwent separate evaluations for in vitro and in vivo experiments, and the comprehensive risk of bias is detailed in Table S4 and Table S5. Notably, three studies featured a combination of in vitro and in vivo assessments, necessitating a comprehensive analysis for the overall risk of bias. For the study conducted by Sun et al., an unclear risk of bias was identified for the in vivo aspect, while a low risk of bias was observed for the in vitro component. Taking both aspects into account, there are uncertainties, thus demonstrating an unclear risk of bias. Similarly, in the study by Wang et al., the overall judgment is deemed unclear due to an uncertain risk of bias identified in the in vivo study. In the study conducted by Flemming et al., numerous domains exhibited an unclear risk of bias, leading to the identification of a high risk of bias for the in vivo aspect. Despite the in vitro aspect displaying a low risk of bias, the overall assessment indicates a high risk of bias.

Supplementary References

1. Lewis, S.J.; Gardner, M.; Higgins, J.; Holly, J.M.P.; Gaunt, T.R.; Perks, C.M.; Turner, S.D.; Rinaldi, S.; Thomas, S.; Harrison, S.; et al. Developing the WCRF International/University of Bristol Methodology for Identifying and Carrying out Systematic Reviews of Mechanisms of Exposure–Cancer Associations. *Cancer Epidemiology Biomarkers and Prevention* **2017**, *26*, doi:10.1158/1055-9965.EPI-17-0232.

Table S1. PRISMA 2020 checklist.

Section and Topic	Item #	Checklist item	Location where item is reported
**TITLE**			
Title	1	Identify the report as a systematic review.	1
**ABSTRACT**			
Abstract	2	See the PRISMA 2020 for Abstracts checklist.	1
**INTRODUCTION**			
Rationale	3	Describe the rationale for the review in the context of existing knowledge.	1-2
Objectives	4	Provide an explicit statement of the objective(s) or question(s) the review addresses.	2
**METHODS**			
Eligibility criteria	5	Specify the inclusion and exclusion criteria for the review and how studies were grouped for the syntheses.	9
Information sources	6	Specify all databases, registers, websites, organisations, reference lists and other sources searched or consulted to identify studies. Specify the date when each source was last searched or consulted.	9
Search strategy	7	Present the full search strategies for all databases, registers and websites, including any filters and limits used.	Table S2
Selection process	8	Specify the methods used to decide whether a study met the inclusion criteria of the review, including how many reviewers screened each record and each report retrieved, whether they worked independently, and if applicable, details of automation tools used in the process.	9
Data collection process	9	Specify the methods used to collect data from reports, including how many reviewers collected data from each report, whether they worked independently, any processes for obtaining or confirming data from study investigators, and if applicable, details of automation tools used in the process.	9
Data items	10a	List and define all outcomes for which data were sought. Specify whether all results that were compatible with each outcome domain in each study were sought (e.g. for all measures, time points, analyses), and if not, the methods used to decide which results to collect.	9
	10b	List and define all other variables for which data were sought (e.g. participant and intervention characteristics, funding sources). Describe any assumptions made about any missing or unclear information.	9
Study risk of bias assessment	11	Specify the methods used to assess risk of bias in the included studies, including details of the tool(s) used, how many reviewers assessed each study and whether they worked independently, and if applicable, details of automation tools used in the process.	9, Table S3
Effect measures	12	Specify for each outcome the effect measure(s) (e.g. risk ratio, mean difference) used in the synthesis or presentation of results.	9
Synthesis methods	13a	Describe the processes used to decide which studies were eligible for each synthesis (e.g. tabulating the study intervention characteristics and comparing against the planned groups for each synthesis (item #5)).	9
	13b	Describe any methods required to prepare the data for presentation or synthesis, such as handling of missing summary statistics, or data conversions.	9-10
	13c	Describe any methods used to tabulate or visually display results of individual studies and syntheses.	9-10
	13d	Describe any methods used to synthesize results and provide a rationale for the choice(s). If meta-analysis was performed, describe the model(s), method(s) to identify the presence and extent of statistical heterogeneity, and software package(s) used.	10
	13e	Describe any methods used to explore possible causes of heterogeneity among study results (e.g. subgroup analysis, meta-regression).	X
	13f	Describe any sensitivity analyses conducted to assess robustness of the synthesized results.	X
Reporting bias assessment	14	Describe any methods used to assess risk of bias due to missing results in a synthesis (arising from reporting biases).	9
Certainty assessment	15	Describe any methods used to assess certainty (or confidence) in the body of evidence for an outcome.	9
**RESULTS**			

Section and Topic	Item #	Checklist item	Location where item is reported
Study selection	16a	Describe the results of the search and selection process, from the number of records identified in the search to the number of studies included in the review, ideally using a flow diagram.	2
	16b	Cite studies that might appear to meet the inclusion criteria, but which were excluded, and explain why they were excluded.	2
Study characteristics	17	Cite each included study and present its characteristics.	3
Risk of bias in studies	18	Present assessments of risk of bias for each included study.	Table S4, Table S5
Results of individual studies	19	For all outcomes, present, for each study: (a) summary statistics for each group (where appropriate) and (b) an effect estimate and its precision (e.g. confidence/credible interval), ideally using structured tables or plots.	3
Results of syntheses	20a	For each synthesis, briefly summarise the characteristics and risk of bias among contributing studies.	2-8
	20b	Present results of all statistical syntheses conducted. If meta-analysis was done, present for each the summary estimate and its precision (e.g. confidence/credible interval) and measures of statistical heterogeneity. If comparing groups, describe the direction of the effect.	X
	20c	Present results of all investigations of possible causes of heterogeneity among study results.	X
	20d	Present results of all sensitivity analyses conducted to assess the robustness of the synthesized results.	X
Reporting biases	21	Present assessments of risk of bias due to missing results (arising from reporting biases) for each synthesis assessed.	Table S4, Table S5
Certainty of evidence	22	Present assessments of certainty (or confidence) in the body of evidence for each outcome assessed.	2-8
**DISCUSSION**			
Discussion	23a	Provide a general interpretation of the results in the context of other evidence.	8-9
	23b	Discuss any limitations of the evidence included in the review.	9
	23c	Discuss any limitations of the review processes used.	9
	23d	Discuss implications of the results for practice, policy, and future research.	9
**OTHER INFORMATION**			
Registration and protocol	24a	Provide registration information for the review, including register name and registration number, or state that the review was not registered.	9
	24b	Indicate where the review protocol can be accessed, or state that a protocol was not prepared.	9
	24c	Describe and explain any amendments to information provided at registration or in the protocol.	X
Support	25	Describe sources of financial or non-financial support for the review, and the role of the funders or sponsors in the review.	10
Competing interests	26	Declare any competing interests of review authors.	10
Availability of data, code and other materials	27	Report which of the following are publicly available and where they can be found: template data collection forms; data extracted from included studies; data used for all analyses; analytic code; any other materials used in the review.	10

From: Page MJ, McKenzie JE, Bossuyt PM, Boutron I, Hoffmann TC, Mulrow CD, et al. The PRISMA 2020 statement: an updated guideline for reporting systematic reviews. BMJ 2021;372:n71. doi: 10.1136/bmj.n71

Table S2. Keywords and MeSH terms used in different databases in the literature search.

Pubmed (517)

"Exosomes"[MeSH Terms] OR "Exosomes" OR "Extracellular Vesicles"[MeSH Terms] OR "Extracellular Vesicl*" OR "Microvesicles" OR "Small Extracellular Vesicles" OR "Small Vesicles") AND ("Carcinoma, Squamous Cell"[MeSH Terms] OR "Carcinoma, Squamous Cell" OR "Basal Cell Carcinoma"[MeSH Terms] OR "Basal Cell Carcinoma" OR "Skin Neoplasms"[MeSH Terms] OR "Skin Neoplasms" OR "Cutaneous Squamous Cell Carcinoma" OR "Basal Cell Carcinoma" OR "Non-Melanoma Skin Cancer" OR "Non-Melanoma Skin Tumor" OR "carcinomas"))

COCHRANE (8)

#1	MeSH descriptor: [Extracellular Vesicles] explode all trees
#2	extracellular vesicl*
#3	exosom*
#4	MeSH descriptor: [Carcinoma, Squamous Cell] explode all trees
#5	squamous cell carcinoma
#6	SCC
#7	basal cell carcinoma
#8	BCC
#9	MeSH descriptor: [Skin Neoplasms] explode all trees
#10	(#1 OR #2 OR #3) AND (#4 OR #5 OR #6 OR #7 OR #8 OR #9)

SCOPUS (363)

(TITLE-ABS-KEY ("extracellular vesicles" OR exosomes OR microvesicles OR "small extracellular vesicles" OR "small vesicles") AND ("carcinoma*, squamous cell" OR "carcinoma, basal cell" OR "skin neoplasms" OR "cutaneous squamous cell carcinoma" OR "non-melanoma skin cancer")

Table S3. Modified RoB-2 tool.

Item	Type of bias	Checklist
1	Selection bias	Have the cells been obtained from a validated repository that guarantees cell verification or have the cells been appropriately independently verified?
2	Selection bias	Is the source of the cell lines reported?
3	Selection bias	Were different cell lines from the same cancer type used in the study?
4	Selection bias	Is the sex of the cells reported?
5	Selection bias	Is there information indicating whether testing for mycoplasma infection in cell lines was conducted?
6	Selection bias	Is it clear whether the cell lines used are primary cell cultures or continuous cell lines?
7	Performance bias	Were interventions administered to cells in a consistent and standardized manner throughout the experiment?
8	Performance bias	Are culture conditions comparable between different studies or groups within studies?
9	Performance bias	Have sufficient biological and technical repeats of the experiments been conducted, and were appropriate controls included?
10	Attrition bias (*)	Were data for this outcome available for all cell lines used? (*)
11	Detection bias	Was the method of measuring the outcome appropriate?
12	Detection bias	Is there clear and transparent reporting on how outcomes were assessed in a manner that minimizes potential bias?
13	Reporting bias (*)	Are reports of the study free of selective outcome reporting? (*)
14	Other bias	Was the study apparently free of other problems that could result in high risk of bias?

* Items consistent with the items in the Cochrane Risk of Bias tool.

Table S4. Quality assessment of in vivo studies.

In vivo studies	Q1	Q2	Q3	Q4	Q5	Q6	Q7	Q8	Q9	Q10	Overall risk of bias
Sun et al. (2018) [30]	Yes	Yes	Unclear	Yes	Unclear	Yes	Unclear	Yes	Yes	Yes	Unclear
Wang et al. (2020) [22]	Yes	Yes	Unclear	Yes	Unclear	Yes	Unclear	Yes	Yes	Yes	Unclear
Flemming J. et al (2020) [19]	Yes	Yes	Unclear	Yes	Unclear	Yes	Unclear	Unclear	Yes	Yes	High

Table S5. Quality assessment of in vitro studies.

In vitro studies	Q1	Q2	Q3	Q4	Q5	Q6	Q7	Q8	Q9	Q10	Q11	Q12	Q13	Q14	Overall risk of bias
Overmiller A. et al. (2017) [7]	Yes	Yes	No	No	No	Yes	Yes	Yes	Unclear	Unclear	Yes	Yes	Yes	Yes	High
Chang et al. (2017) [27]	Unclear	Yes	Yes	Yes	No	Yes	Yes	Yes	Unclear	Unclear	Yes	Yes	Yes	Yes	Unclear
Sun et al. (2018)	Yes	Yes	Yes	No	No	Yes	Yes	Yes	Unclear	No	Yes	Yes	Yes	Yes	Low
Zhao Z. et al. (2020) [18]	Yes	Yes	Yes	No	No	Yes	Yes	Yes	Unclear	Yes	Yes	Yes	Yes	Yes	Low
Wang et al. (2020) [22]	Yes	Yes	Yes	No	No	Yes	Yes	Yes	Yes	Yes	Yes	Yes	Yes	Yes	Low
Flemming J. et al (2020) [19]	Yes	Yes	Yes	No	No	Yes	Yes	Yes	Unclear	Yes	Yes	Yes	Yes	Yes	Low
Zhang Z. et al. (2021) [3]	Yes	Yes	Yes	No	No	Yes	Yes	Yes	Unclear	Yes	Yes	Yes	Yes	Yes	Low
Zauner R et al. (2023) [31]	No	Yes	Yes	Yes	No	Yes	Yes	Yes	Unclear	No	Yes	Yes	Yes	Yes	High
